# Heyde syndrome: prevalence and outcomes in patients undergoing transcatheter aortic valve implantation

**DOI:** 10.1007/s00392-021-01905-z

**Published:** 2021-07-23

**Authors:** Lara Waldschmidt, Andreas Drolz, Paula Heimburg, Alina Goßling, Sebastian Ludwig, Lisa Voigtländer, Matthias Linder, Niklas Schofer, Hermann Reichenspurner, Stefan Blankenberg, Dirk Westermann, Lenard Conradi, Johannes Kluwe, Moritz Seiffert

**Affiliations:** 1 Department of Cardiology, University Heart and Vascular Center Hamburg, Universitätsklinikum Hamburg-Eppendorf (UKE), Martinistrasse 52, 20246 Hamburg, Germany; 2 Department of Cardiovascular Surgery, University Heart and Vascular Center Hamburg, Hamburg, Germany; 3 Department of Gastroenterology, University Hospital Hamburg-Eppendorf, Hamburg, Germany

**Keywords:** TAVI, Heyde syndrome, Gastrointestinal bleeding, Angiodysplasia

## Abstract

**Background:**

Heyde syndrome (HS) is known as the association of severe aortic stenosis (AS) and recurrent gastrointestinal bleeding (GIB) from angiodysplasia. Data on the prevalence of HS and results after TAVI remain scarce.

**Methods:**

2548 consecutive patients who underwent TAVI for the treatment of AS from 2008 to 2017 were evaluated for a history of GIB and the presence of HS. The diagnosis of HS was defined as a clinical triad of severe AS, a history of recurrent GIB, and an endoscopic diagnosis of angiodysplasia. These patients (Heyde) were followed to investigate clinical outcomes, bleeding complications and the recurrence of GIB and were compared to patients with GIB unrelated to HS (Non-Heyde).

**Results:**

A history of GIB prior to TAVI was detected in 190 patients (7.5%). Among them, 47 patients were diagnosed with HS (1.8%). Heyde patients required blood transfusions more frequently compared to Non-Heyde patients during index hospitalization (50.0% vs. 31.9%, *p* = 0.03). Recurrent GIB was detected in 39.8% of Heyde compared to 21.2% of Non-Heyde patients one year after TAVI (*p* = 0.03). In patients diagnosed with HS and recurrent GIB after TAVI, the rate of residual ≥ mild paravalvular leakage (PVL) was higher compared to those without recurrent bleeding (73.3% vs. 38.1%, *p* = 0.05).

**Conclusion:**

A relevant number of patients undergoing TAVI were diagnosed with HS. Recurrent GIB was detected in a significant number of Heyde patients during follow-up. A possible association with residual PVL requires further investigation to improve treatment options and outcomes in patients with HS.

**Graphic abstract:**

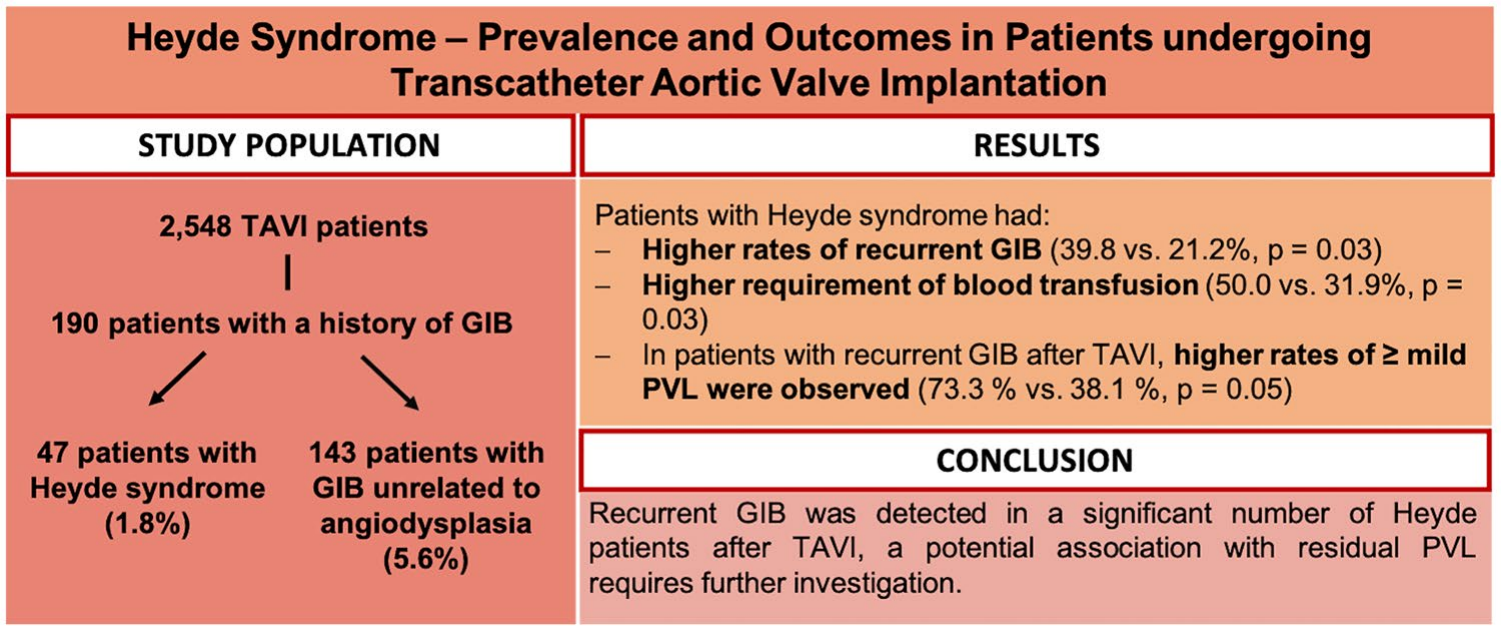

**Supplementary Information:**

The online version contains supplementary material available at 10.1007/s00392-021-01905-z.

## Introduction

The association of aortic valve stenosis (AS) and gastrointestinal bleeding (GIB) from angiodysplasia is known as Heyde syndrome (HS) [1]. The prevalence of GIB related to HS has been described as 1 to 3 percent of patients with aortic stenosis [2–5].

The prevalence, pathogenesis and treatment options of HS remain controversial. The development of angiodysplasia has been associated with mucosal ischemia leading to vessel dilatation and formation of coral-formed vessels throughout the GI tract with highest prevalence in the right colon and cecum [6]. The pathogenesis of HS involves hemostaseologic alterations leading to an acquired von Willebrand syndrome (aVWS). High shear stress caused by severe aortic stenosis leads to a decreased content of the high-molecular-weight multimers (HMWM) and decreased collagen-binding activity of VWF [7–9]. The combination of both factors, vascular malformations, i.e. angiodysplasia, and hemostaseologic alterations due to AS, result in a higher bleeding risk in these patients [10].

Surgical or transcatheter implantation of an aortic valve prosthesis is commonly performed for the treatment of severe AS. A resolution of GIB from angiodysplasia in patients with HS by surgical aortic valve replacement (SAVR) in the majority of patients has been previously reported [11, 12]. Since transcatheter aortic valve implantation (TAVI) has become the preferred treatment in the majority of patients with severe AS, it remains to be determined whether TAVI would likewise resolve recurrent GIB in patients with HS. First reports suggested a resolution of bleeding but these assumptions were drawn from small analyses including only few Heyde patients or case reports and evidence overall remains scarce [3, 4, 8, 13–16].

Thus, we aimed to evaluate the prevalence of HS in a real-life cohort of patients undergoing TAVI, to identify variables associated with bleeding events in HS and to analyze the incidence of recurrent GIB during post-TAVI follow-up in these patients.

## Methods

### Study population

We conducted a retrospective single-center analysis of 2548 consecutive patients who underwent TAVI for the treatment of severe AS at a single academic heart center between 2008 and 2017. The diagnosis of severe AS was made according to the current ESC/EACTS guidelines for the treatment of valvular heart disease [17]. Among all patients treated with TAVI at our institution, those with a history of GIB were identified. A history of GIB was defined as an episode of GIB recorded in a patient’s chart, earlier diagnoses or endoscopy reports. The diagnosis of HS was based on the clinical triad of severe AS, a history of recurrent GIB, and an endoscopic (esophagogastroduodenoscopy or colonoscopy) diagnosis of angiodysplasia. GIB of unknown origin or related to other causes (e.g. malignant process, ulcer) was defined as bleeding unrelated to angiodysplasia. We collected baseline and follow-up data of both groups (a) Heyde patients (*n* = 47) and (b) those with GIB unrelated to angiodysplasia (Non-Heyde patients, *n* = 143).

### Patient follow-up

Baseline and follow-up variables were recorded from patients’ charts and entered into a dedicated database. Mean follow-up was 11.2 months. Clinical outcomes were evaluated with emphasis on bleeding complications and recurrence of GIB. Clinical endpoints and periprocedural complications were defined in accordance with the updated Valve Academic Research Consortium-2 (VARC-2) definitions [18]. Endpoint adjudication was performed independently from this study by a team of experienced cardiologists who were blinded to the study groups.

### Statistical analyses

Continuous variables were shown as median (25th and 75th percentile) or as mean ± standard deviation. Binary variables or absolute and relative frequencies were shown as number (*n*) and percentage. Differences to total n were due to missing values, the calculation of proportions did not include missing values in the denominator. For two group comparison Mann–Whitney test was used for continuous variables, and χ^2^ test for binary ones. Survival curves for GIB after TAVI during 1-year follow-up were produced using the Kaplan–Meier method. Survival curve differences were tested using the log-rank test. For overall tests p < 0.05 was considered statistically significant. All statistical analyses were performed using R version 4.0.3 (R Foundation for Statistical Computing).

## Results

### Baseline characteristics

**Fig. 1:**
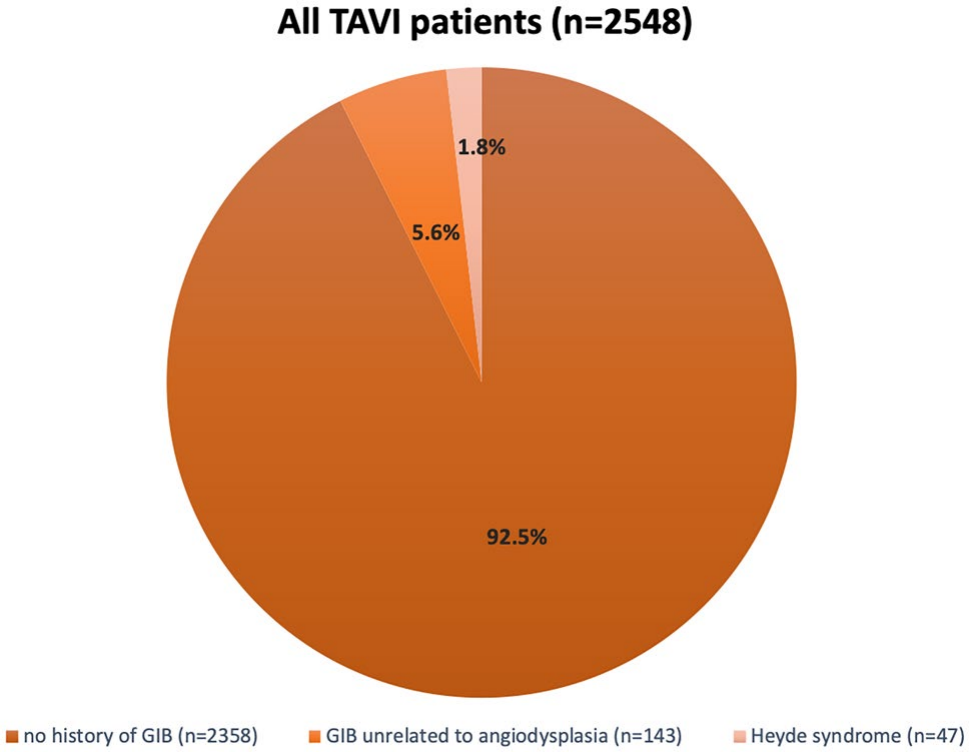
Distribution of patients with GIB among all patients with severe AS treated with TAVI

The median age of all patients undergoing TAVI (*n* = 2548) was 81.2 years (76.4, 85.0) and 49.3% were male. Among these patients, a total of 190 patients (7.5%) had a history of GIB. Overall 47 patients (1.8%) fulfilled the clinical characteristics for HS (history of recurrent GIB and an endoscopic diagnosis of angiodysplasia of the GI tract), while 143 patients (5.6%) had a history of GIB unrelated to angiodysplasia (Non-Heyde patients, see Fig. 1). Both groups were similar with regard to age, gender, comorbidities, and risk profiles (see Table 1). Antithrombotic regimens in Heyde vs. Non-Heyde patients did not differ significantly at baseline (single antiplatelet: 44.4% vs. 50.0%, *p* = 0.61; dual antiplatelet: 8.9% vs. 6.5%, *p* = 0.74; oral anticoagulation: 28.9% vs. 39.9%, *p* = 0.22). Hemoglobin levels were lower in patients with HS compared to those with GIB unrelated to angiodysplasia (all results as follows Heyde vs. Non-Heyde, Hb 10.0 (8.7,11.1) g/dl vs. 10.8 (9.4,12.2) g/dl; *p* = 0.02, see Table 1). The number of patients with hemoglobin concentrations < 8 g/dl was numerically higher in Heyde vs. Non-Heyde, albeit not statistically significant.

**Table 1:** Baseline characteristics and comorbidities in all TAVI patients and patients with Heyde syndrome compared to patients with GIB unrelated to angiodysplasia (Non-Heyde)

	All (*n* = 2548)	Heyde (*n* = 47)	Non-Heyde (*n* = 143)	*p* Value
Baseline data				
Age (years)	81.2 (76.4,85.0)	80.7 (75.3,84.0)	80.1 (74.2,84.2)	0.68
Male (%)	1255 (49.3)	23 (48.9)	74 (51.7)	0.87
STS PROM (%)	4.9 (3.2,7.6)	4.7 (2.7,9.0)	5.3 (3.6,8.3)	0.39
Atrial fibrillation (%)	783 (31.8)	21 (44.7)	73 (54.1)	0.31
Arterial hypertension (%)	2132 (84.6)	39 (83.0)	130 (90.9)	0.18
Diabetes (%)	732 (29.0)	16 (34.0)	45 (31.5)	0.86
CAD (%)	1640 (64.9)	29 (61.7)	105 (73.9)	0.14
Prior PCI (%)	904 (35.7)	18 (38.3)	53 (37.3)	1.00
COPD (%)	520 (20.4)	11 (23.4)	32 (22.4)	1.00
Hemoglobin (g/dl)	11.9 (10.6,13.1)	10.0 (8.7,11.1)	10.8 (9.4,12.2)	0.02
Hemoglobin < 8 g/dl (%)	28 (1.1)	5 (10.6)	6 (4.2)	0.14
GFR (CKD-EPI) (ml/min/1.73m^2^)	57.2 (40.8,74.9)	54.1 (37.1,70.8)	49.5 (36.4,67.8)	0.31
LVEF < 30% (%)	264 (10.6)	2 (4.3)	13 (9.4)	0.36
EOA AV (cm^2^)	0.8 (0.6, 0.9)	0.9 (0.7,0.9)	0.8 (0.6,0.9)	0.10
Pmean (mmHg)	32 (22.0, 45.0)	33.0 (23.7, 45.3)	36.0 (24.1, 47.0)	0.56

Values are n (%), mean $\pm$ SD or median (interquartile range). *P* values are Heyde vs. Non-Heyde

*AV*  aortic valve, *CAD* coronary artery disease, *COPD* chronic obstructive pulmonary disease, *EOA* effective orifice area, *GFR* glomerular filtration rate, *LVEF* left ventricular ejection fraction, *Pmean* mean pressure gradient, *STS PROM* Society of Thoracic Surgeons Predicted Risk of Mortality

### Periprocedural data

TAVI was performed with immediate technical success in all cases (*n* = 190), most frequently by endovascular access (68.1% vs. 76.2%, p = 0.34). Effective orifice areas (EOA) increased from 0.8 ± 0.2 cm^2^ to 2.1 ± 0.5 cm^2^ overall and similar in both groups. VARC-2 defined complications including major access site complications (12.8% vs. 9.1%, *p* = 0.57), any stroke or TIA (4.8% vs. 4.2%, *p* = 1.00) and permanent pacemaker implantations (16.7% vs. 20.3%, p = 0.66) were similar in both groups. We observed a trend to more acute renal failure (AKIN ≥ 2) in Heyde vs. Non-Heyde patients (9.5% vs. 2.5%, *p* = 0.08). Periprocedural major or life-threatening bleeding events were numerically higher in Heyde vs. Non-Heyde patients, albeit not statistically significant (21.3% vs. 13.4%, *p* = 0.24). Among these patients GIB occurred more often in patients with HS during 30-day follow-up (19.4 vs. 7.0%, *p* = 0.05, see Table 2). The incidence of GIB in the overall TAVI cohort was 1.5%. 50.0% of Heyde patients required transfusion of 2.3 $\pm$ 4.6 packed red blood cells (PRBC) during the index hospitalization compared to 1.5 $\pm$ 4.0 PRBC in 31.9% of patients with a history of GIB unrelated to angiodysplasia (Non-Heyde) (*p* = 0.03, see Table 2).

**Table 2:** Periprocedural and discharge data in patients with Heyde syndrome compared to patients with GIB unrelated to angiodysplasia (Non-Heyde)

	All (*n* = 2548)	Heyde (*n* = 47)	Non-Heyde (*n* = 143)	*p* Value
Periprocedural Data				
Endovascular access (%)	1721 (67.5)	32 (68.1)	109 (76.2)	0.34
Balloon-expandable devices* (%)	1206 (47.3)	27 (57.4)	72 (50.3)	0.41
Self-expandable devices^a^ (%)	1180 (46.3)	16 (34.0)	67 (46.9)	0.13
Mechanically expandable devices^b^ (%)	130 (5.1)	4 (8.5)	4 (2.8)	0.11
Discharge Data and Follow-Up according to VARC-2 at 30 days				
Major access site complications (%)	194 (7.7)	6 (12.8)	13 (9.1)	0.57
Acute renal failure (AKIN ≥ 2) (%)	131 (6.1)	4 (9.5)	3 (2.5)	0.08
Any stroke or TIA (%)	105 (4.9)	2 (4.8)	5 (4.2)	1.00
Permanent pacemaker implantation (%)	386 (17.8)	7 (16.7)	24 (20.3)	0.66
Bleeding VARC (major/life-threatening) (%)	291 (13.3)	10 (21.3)	19 (13.4)	0.24
Proportion of GIB (%)^c^	32 (1.5)	7 (19.4)	7 (7.0)	0.05
Transfusion of PRBC (%)	737 (30.2)	23 (50.0)	45 (31.9)	0.03
Number of PRBC transfusions during indexstay	1.2 $\pm$ 3.3	2.3 $\pm$ 4.6	1.5 $\pm$ 4.0	0.03
Single antiplatelet therapy (SAPT) (%)	356 (15.1)	12 (30.0)	30 (22.6)	0.40
Dual antiplatelet therapy (DAPT) (%)	850 (36.1)	15 (37.5)	46 (34.6)	0.85
(O)AC (%)	111 (4.7)	1 (2.5)	8 (6.0)	0.69
(O)AC + SAPT (%)	663 (28.2)	10 (25.0)	40 (30.1)	0.69
(O)AC + DAPT (%)	339 (14.4)	2 (5.0)	9 (6.8)	1.00
Duration of intensified antithrombotic therapy (months)^d^	1.0 (0, 3.0)	1.0 (0, 3.0)	1.5 (0, 3.0)	0.61
EOA AV (cm^2^)	1.7 (1.4, 2.1)	2.3 (1.7, 2.5)	2.3 (1.8, 2.5)	0.55
Pmean (mmHg)	9.0 (6.0, 12.7)	9.0 (6.0, 14.1)	9.0 (6.0, 12.0)	0.34
PVL ≥ mild (%)	1012 (44.4)	25 (54.3)	77 (56.2)	0.86
Hemoglobin (g/dl)	9.4 (8.7,10.3)	9.5 (8.8,10.2)	9.3 (8.7,10.4)	0.87
GFR (CKD-EPI) (ml/min/1.73m^2^)	56.3 (38.5,60.0)	56.8 (38.5,60.0)	56.2 (38.5,60.0)	0.71
30-day mortality (%)	185 (7.3)	4 (8.5)	8 (5.6)	0.50
1-year mortality (%)	535 (21.2)	13 (27.7)	33 (23.1)	0.56

Values are *n* (%), mean $\pm$ SD or median (interquartile range). *P* values are Heyde vs. Non-Heyde

^*^SAPIEN XT, 3, Ultra

^a^Evolut R, Portico, ACURATE neo, ALLEGRA

^b^Lotus (edge).

^c^Proportion of GIB within VARC-2 major/life-threatening bleeding at 30 days *DAPT* dual antiplatelet therapy, *EOA* effective orifice area, *GFR* glomerular filtration rate, *GIB* gastrointestinal bleeding, *LMH* low molecular heparin, *LVEF* left ventricular ejection fraction, (O)AC (oral) anticoagulants (Phenprocoumon, new anticoagulants, (low molecular weight) heparin), *Pmean* mean pressure gradient, *PCI* percutaneous coronary intervention, *PRBC* packed red blood cells, *PVL* paravalvular leakage, *SAPT* single antiplatelet therapy, *VARC* Valvular Academic Research Consortium

^d^Duration of DAPT or (O)AC + SAPT, (O)AC + DAPT.

### Recurrence of GI-bleeding during follow-up

**Fig. 2:**
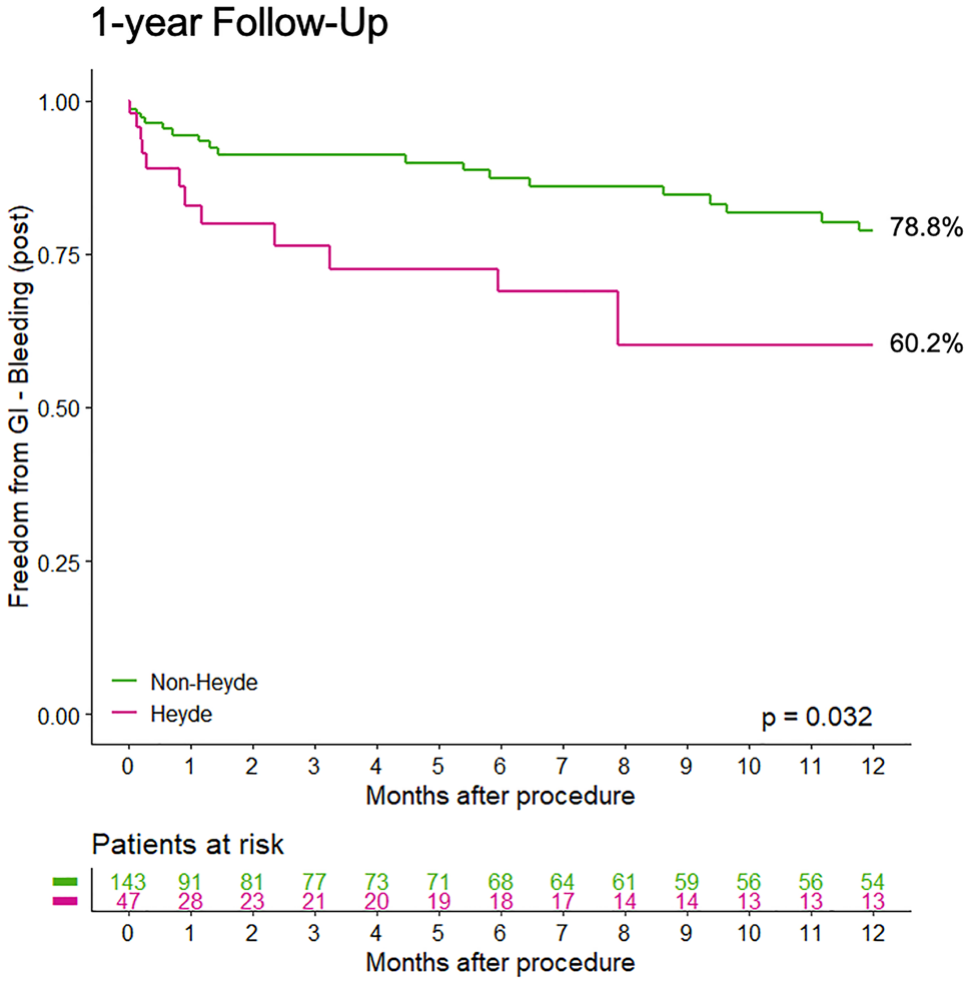
Freedom from GIB in patients with Heyde syndrome compared to those with GIB unrelated to angiodysplasia (Non-Heyde) during 1 year of follow-up (post TAVI). Significantly higher GI-bleeding complications in patients with Heyde syndrome during 1 year of follow-up were revealed (*p* = 0.03)

Freedom from GIB after TAVI during 1-year follow-up was significantly lower in Heyde patients (60.2%) compared to patients with GIB unrelated to angiodysplasia (Non-Heyde, 78.8%; *p* = 0.03, see Fig. 2). During the follow-up period 15 (44%) patients with Heyde syndrome compared to 26 (26%) of Non-Heyde patients had recurrent GIB (*p* = 0.02, see **table S1**, Supplementary Material). A total of 9 (60%) Heyde patients had a diagnosis of upper or lower GIB, 6 of whom had an endoscopic diagnosis of angiodysplasia. In the remaining 9 Heyde patients there was either no evidence of angiodysplasia or endoscopy was not completed. In Non-Heyde patients 11 (42%) had a diagnosis of upper of lower GIB during follow-up and 15 (58%) had GIB of unknown origin. The source of bleeding was multifactorial (e.g. ulcers, gastritis) but angiodysplasia was found in two cases, one of whom had moderate PVL at the time of endoscopy.

Antithrombotic and anticoagulation regimens after TAVI were similar among Heyde vs. Non-Heyde patients (see Table 2). The duration of intensified antithrombotic therapy (i.e. combination of (oral) anticoagulation (OAC) and single or dual antiplatelet therapy (SAPT/DAPT)) did not differ between both groups. Antithrombotic strategies did not differ in HS patients with and without recurrent bleeding during follow-up. A trend towards higher rates of intensified antithrombotic therapy (OAC + SAPT or DAPT) was observed in Non-Heyde patients with recurrent GIB (see **figure S2**, Supplementary Material).

**Fig. 3:**
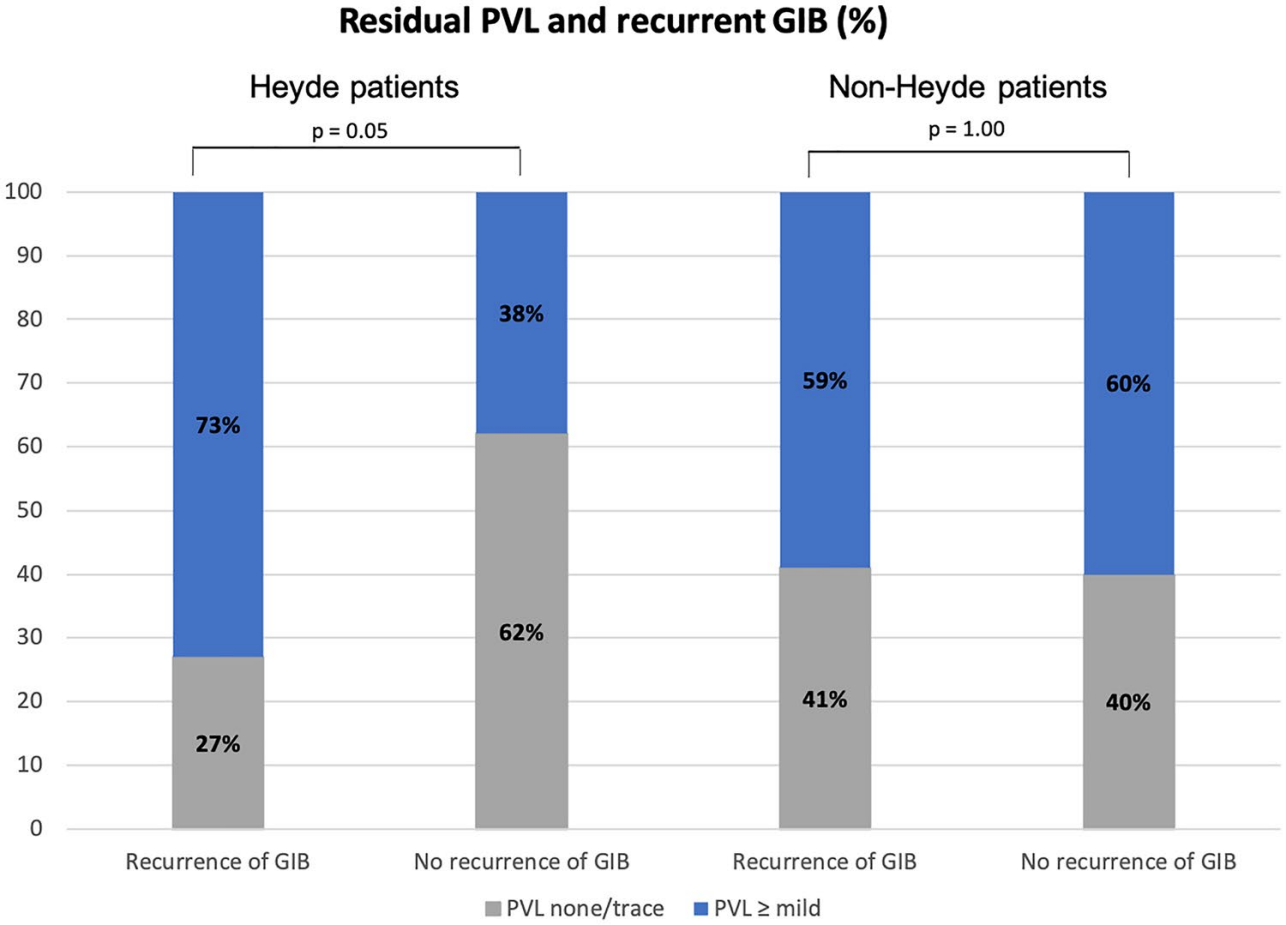
Distribution of paravalvular leakage (PVL) in patients with and without Heyde syndrome with regard to recurrence of gastrointestinal bleeding during follow-up

In patients diagnosed with HS and recurrent GIB after TAVI the rate of residual mild or moderate paravalvular regurgitation was higher compared to those with an unremarkable course while statistically this difference reached borderline significance (73.3% vs. 38.1%, *p* = 0.05, see Fig. 3).

## Discussion

In the present study we assessed the prevalence of HS in a real-life cohort of patients with severe AS undergoing TAVI and evaluated bleeding complications and GIB during follow-up.

Three key findings can be drawn from this retrospective single-center analysis: First, HS was prevalent in a relevant number of TAVI patients (1.8%) which was in line with previously published data from smaller observational analyses [2–5]. To date, the present study is the largest cohort of TAVI patients evaluating the prevalence of HS. Second, during a follow-up period of 12 months after TAVI the recurrence of GIB in Heyde patients was significant (39.8%) and higher compared to patients with GIB unrelated to angiodysplasia. Third, patients with HS and recurrent GIB during follow-up had more residual paravalvular regurgitation compared to Heyde patients with an unremarkable course.

Previous studies reported a resolution of GIB from angiodysplasia after SAVR in the majority of Heyde patients, although systematic evidence was lacking [11, 12, 19–22]. Hemostaseologic findings with a correction of the HMWM content after SAVR suggested a “surgical cure” of HS [9, 23]. Within the past decade, TAVI has become the preferred treatment option for AS in most patients [24, 25]. A normalization of hemostasis parameters including vWF was reported after TAVI, especially in those with pre-existing aVWS [4, 8]. Few small studies and case series reported no recurrence of GIB in patients with HS after TAVI, albeit limited due to short-term follow-up and small patient numbers [3, 4, 8, 13–15]. In contrast to these earlier publications, we observed that a substantial number of Heyde patients suffered from recurrent GIB after TAVI already during 1-year follow-up despite adequate treatment of AS.

An association of paravalvular leakage (PVL), late bleeding complications and impaired survival was demonstrated before. An analysis from the PARTNER (Placement of Aortic Transcatheter Trial) trial found late bleeding events, mainly GIB, in a relevant number of patients (5.9%) after TAVI, which were associated with a fourfold increase in late mortality (26). Interestingly, PVL was the strongest predictor of these bleeding events between 30 days and 1 year after TAVI [26]. Moreover, mild to moderate PVL was discovered in patients without recovery of abnormal multimer levels, emphasizing the effect of persistent shear stress as cause of HMWM deficiency [8] Consistent with these findings, we discovered a significantly higher rate of ≥ mild PVL in Heyde patients with recurrent GIB after TAVI compared to those with an unremarkable course. As angiodysplasias persist after replacement of the aortic valve [2] this suggests that the association between PVL and recurrent GIB is caused by hemostaseologic rather than vascular alterations associated by PVL. Future studies should evaluate if individualized antithrombotic strategies, e.g. guided by post-TAVI HMWM levels or vWF activity can prevent GIB events in patients with HS (with PVL) after TAVI.

Furthermore, we found more periprocedural transfusion of packed red blood cells in Heyde patients, possibly related to lower baseline hemoglobin values and numerically higher rates of bleeding complications. The adverse impact of red blood cell transfusion after TAVI was previously described [27–29], emphasizing the need for close monitoring of this vulnerable patient cohort.

Individualized antithrombotic treatment will be essential to minimize bleeding risk in these patients at risk. Recent evidence demonstrated superiority for restrictive antithrombotic strategies after TAVI [30, 31]. Additional analyses suggested that patients after TAVI may have hemostatic disorders apart from aVWS that may cause bleeding complications [32]. Whether bleeding complications can be reduced with tailored antithrombotic strategies in these vulnerable HS patients, needs further investigation.

Limitations of the current study relate to the retrospective single-center study design. Since TAVI patients from 2008 to 2017 were included, early data include the learning curve, high rates of non-transfemoral access, paravalvular regurgitation and notable rates of short- and mid-term mortality compared to current practice. Despite careful evaluation of clinical documentation and discharge letters, incomplete detection of bleeding episodes cannot entirely be ruled out. Our focus was on the evaluation of clinical bleeding events rather than hemostaseologic parameters. Hence, levels of vWF were not be included in this analysis. Additional prospective studies with larger patient samples may address these shortcomings in the future.

## Conclusion

In this large cohort, we demonstrated that Heyde syndrome was prevalent in a relevant number of patients presenting for the treatment of AS. In contrast to earlier publications, a substantial number of recurrent GIB events was observed despite correction of AS by TAVI. Patients with residual paravalvular regurgitation after TAVI may be at higher risk of GIB and should be monitored more closely for bleeding events. In the meantime, optimal hemodynamic results and individualized antithrombotic strategies should be targeted to minimize bleeding risk and improve outcomes in these vulnerable patients with Heyde syndrome.

## Supplementary Information

Below is the link to the electronic supplementary material.

Supplementary file1 (PNG 206 KB)

Supplementary file2 (PNG 91 KB)

## Abbreviations

AS: Aortic stenosis
GI: Gastrointestinal
GIB: Gastrointestinal bleeding
HS: Heyde syndrome
PVL: Paravalvular leak
SAVR: Surgical aortic valve replacement
STS PROM: Society for Thoracic Surgeons—predicted risk of mortality
TAVI: Transcatheter aortic valve implantation
VWF: Von Willebrand factor
aVWS: Acquired von Willebrand syndrome

## References

[1] HEYDE., C. E. Gastrointestinal bleeding in aortic stenosis (letter). N Engl J Med 1958;259(4):196.

[2] Undas A, Natorska J (2015). Bleeding in patients with severe aortic stenosis in the era of transcatheter aortic valve replacement. JACC Cardiovasc Interv.

[3] Godino C, Lauretta L, Pavon AG (2013). Heyde’s syndrome incidence and outcome in patients undergoing transcatheter aortic valve implantation. J Am Coll Cardiol.

[4] Caspar T, Jesel L, Desprez D (2015). Effects of transcutaneous aortic valve implantation on aortic valve disease-related hemostatic disorders involving von Willebrand factor. Can J Cardiol.

[5] Desai R, Parekh T, Singh S (2019). Alarming increasing trends in hospitalizations and mortality with heyde’s syndrome: a nationwide inpatient perspective (2007 to 2014). Am J Cardiol.

[6] Sharma R, Gorbien MJ (1995). Angiodysplasia and lower gastrointestinal tract bleeding in elderly patients. Arch Intern Med.

[7] Le TT, Susen S, Caron C (2008). Functional impairment of Von Willebrand factor in hypertrophic cardiomyopathy relation to rest and exercise obstruction. Circulation.

[8] Spangenberg T, Budde U, Schewel D (2015). Treatment of acquired von willebrand syndrome in aortic stenosis with transcatheter aortic valve replacement. JACC Cardiovasc Interv.

[9] Vincentelli A, Susen S, Le Tourneau T (2003). Acquired von Willebrand syndrome in aortic stenosis. N Engl J Med.

[10] Warkentin TE, Moore JC, Anand SS, Lonn EM, Morgan DG (2003). Gastrointestinal bleeding, angiodysplasia, cardiovascular disease, and acquired von Willebrand syndrome. Transfus Med Rev.

[11] Thompson JL, Schaff HV, Dearani JA (2012). Risk of recurrent gastrointestinal bleeding after aortic valve replacement in patients with Heyde syndrome. J Thorac Cardiovasc Surg.

[12] Abi-Akar R, El-Rassi I, Karam N, Jassar Y, Slim R, Jebara V (2011). Treatment of heyde’s syndrome by aortic valve replacement. Curr Cardiol Rev.

[13] Ramachandran R, Uqdah H, Jani N (2018). A case of recurrent obscure gastrointestinal bleeding: Heyde’s syndrome - case report and review. J Community Hosp Intern Med Perspect.

[14] Pozzi M, Hanss M, Petrosyan A (2014). Resolution of acquired von Willebrand syndrome after transcatheter aortic valve implantation through a left transcarotid approach. Int J Cardiol.

[15] Balbo CP, Seabra LP, Galoro VG (2016). Heyde’s syndrome and transcatheter aortic valve implantation. Arq Bras Cardiol.

[16] Sedaghat A, Kulka H, Sinning J-M (2017). Transcatheter aortic valve implantation leads to a restoration of von Willebrand factor (VWF) abnormalities in patients with severe aortic stenosis – Incidence and relevance of clinical and subclinical VWF dysfunction in patients undergoing transfemoral TAVI. Thromb Res.

[17] Baumgartner H, Falk V, Bax JJ (2018). 2017 ESC/EACTS Guidelines for the Management of Valvular Heart Disease. Rev Esp Cardiol (Engl Ed).

[18] Kappetein AP, Head SJ, Généreux P (2012). Updated standardized endpoint definitions for transcatheter aortic valve implantation. J Am Coll Cardiol.

[19] Love JW (1982). The syndrome of calcific aortic stenosis and gastrointestinal bleeding: resolution following aortic valve replacement. J Thorac Cardiovasc Surg.

[20] Boyle JM, Rowen HE, Saito H, Vicic WJ, Ankeney JL (1981). Severe aortic stenosis in a patient with recurrent gastrointestinal bleeding: replacement of the aortic valve with a porcine xenograft. Am J Gastroenterol.

[21] King RM, Pluth JR, Giuliani ER (1987). The association of unexplained gastrointestinal bleeding with calcific aortic stenosis. Ann Thorac Surg.

[22] Anderson RP, McGrath K, Street A (1996). Reversal of aortic stenosis, bleeding gastrointestinal angiodysplasia, and von Willebrand syndrome by aortic valve replacement. Lancet (London, England).

[23] Warkentin TE, Moore JC, Morgan DG (2002). Gastrointestinal angiodysplasia and aortic stenosis. N Engl J Med.

[24] Mack MJ, Leon MB, Thourani VH (2019). Transcatheter aortic-valve replacement with a balloon-expandable valve in low-risk patients. N Engl J Med.

[25] FDA expands indication for several transcatheter heart valves to patients at low risk for death or major complications associated with open-heart surgery | FDA. Available at: https://www.fda.gov/news-events/press-announcements/fda-expands-indication-several-transcatheter-heart-valves-patients-low-risk-death-or-major. Accessed January 10, 2021.

[26] Généreux P, Cohen DJ, Mack M (2014). Incidence, predictors, and prognostic impact of late bleeding complications after transcatheter aortic valve replacement. J Am Coll Cardiol.

[27] Généreux P, Cohen DJ, Williams MR (2014). Bleeding complications after surgical aortic valve replacement compared with transcatheter aortic valve replacement: Insights from the PARTNER i trial (Placement of Aortic Transcatheter Valve). J Am Coll Cardiol.

[28] Pilgrim T, Stortecky S, Luterbacher F, Windecker S, Wenaweser P (2013). Transcatheter aortic valve implantation and bleeding: Incidence, predictors and prognosis. J Thromb Thrombolysis.

[29] Seiffert M, Conradi L, Terstesse AC (2015). Blood transfusion is associated with impaired outcome after transcatheter aortic valve implantation. Catheter Cardiovasc Interv.

[30] Brouwer J, Nijenhuis VJ, Delewi R (2020). Aspirin with or without clopidogrel after transcatheter aortic-valve implantation. N Engl J Med.

[31] Nijenhuis VJ, Brouwer J, Delewi R (2020). Anticoagulation with or without clopidogrel after transcatheter aortic-valve implantation. N Engl J Med.

[32] Dauerman HL, DeStephan CM, Sommer HT (2019). Prolonged clotting time among patients undergoing transcatheter aortic valve replacement. J Am Coll Cardiol.

