# Supplementary figures and images for: Heyde syndrome: prevalence and outcomes in patients undergoing transcatheter aortic valve implantation
Source: Clin Res Cardiol. 2021 Jul 23;110(12):1939–46. doi: 10.1007/s00392-021-01905-z (PMC8639542; doi:10.1007/s00392-021-01905-z)

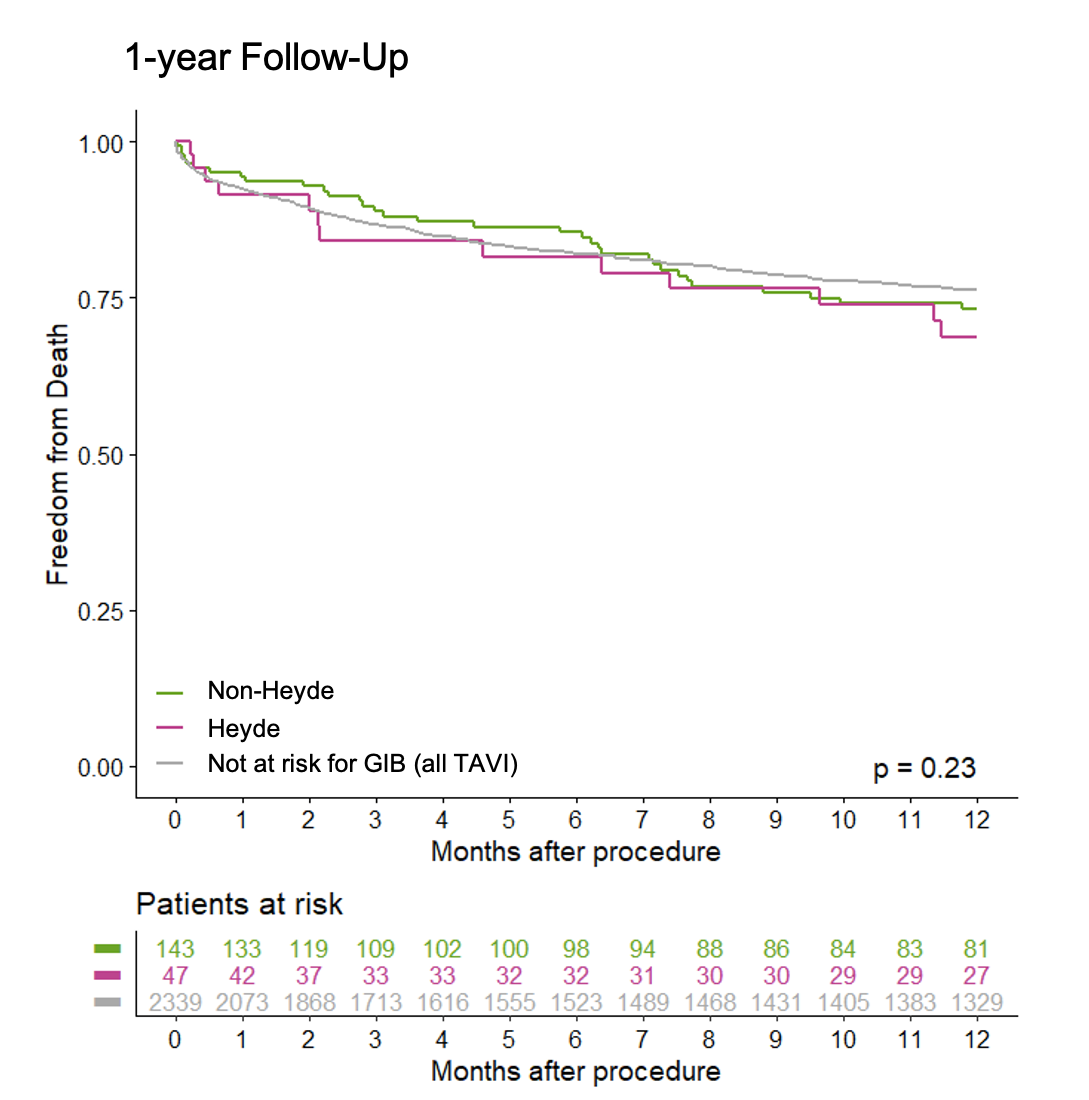

Supplement: Supplementary file 1 — Supplementary file1 (PNG 206 KB) [file 392_2021_1905_MOESM1_ESM.png]

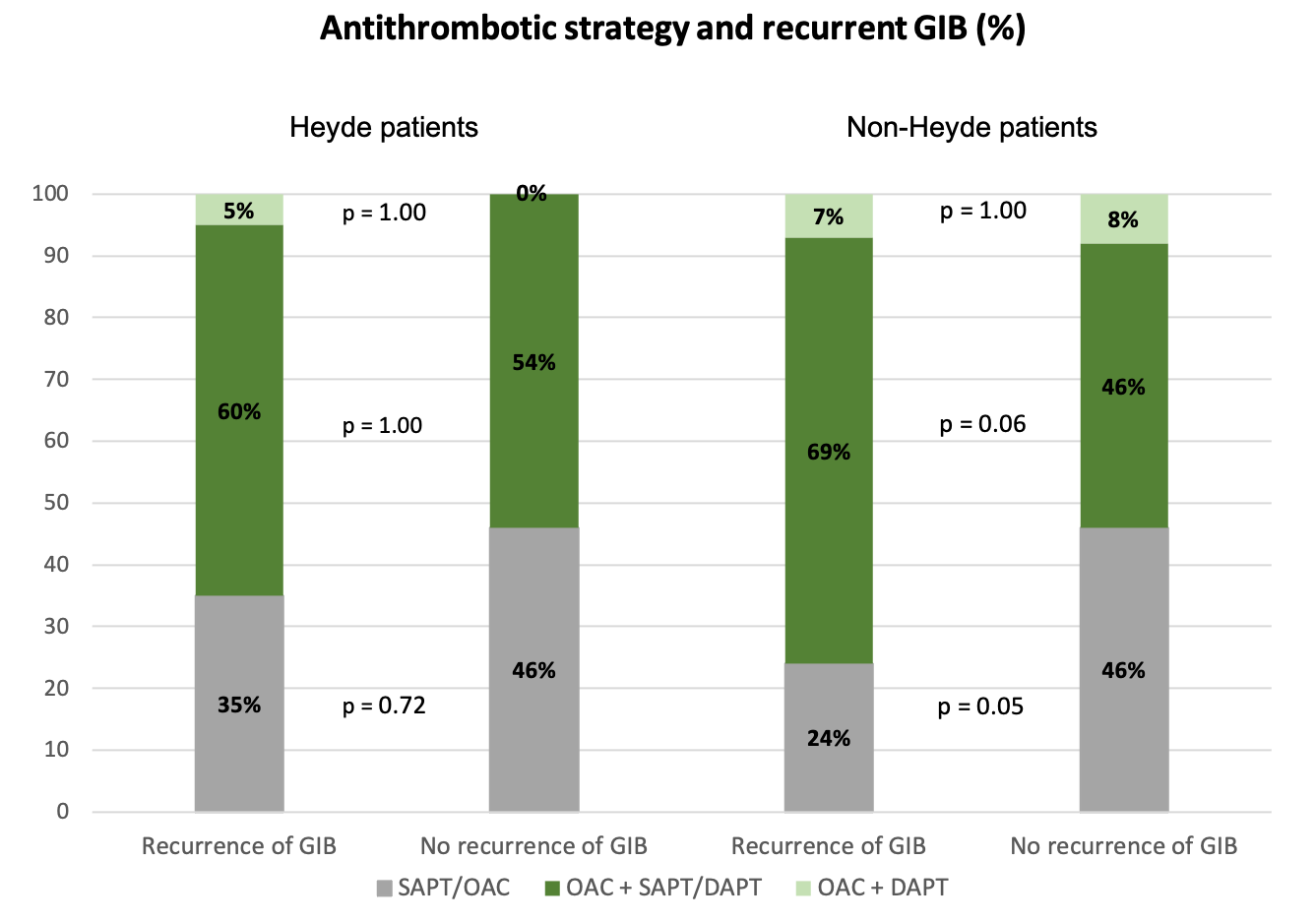

Supplement: Supplementary file 2 — Supplementary file2 (PNG 91 KB) [file 392_2021_1905_MOESM2_ESM.png]
